# Selections and Abstracts

**Published:** 1908-03

**Authors:** 


### SELECTIONS AND ABSTRACTS.

### TREATMENT OF MALARIA.

---

Paine thus outlines the management of malaria:

Treatment of the Paroxysm.—If the physician arrives before the chill has occurred, a hypodermic injection of one-fourth grain of morphine sulphate and 1-150 of a grain of atropine sulphate will markedly modify the severity of the chill. During the chill itself, the patient may be placed in bed, bundled up in warm blankets, given hot drinks, such as hot lemonade or whisky, and have hot water bottles applied to the extremities. The chill soon passes off, however, and is replaced by a burning fever, which may last several hours and is best treated by tepid sponge baths, ice bag to the head, cool drinks and small doses of phenacetin or acetanilid, three or four grains of the former or two grains of the latter every two hours. Finally the fever passes away and the patient becomes bathed in a profuse perspiration which constitutes the sweating stage. All that is necessary to do now is to change the under garments and clothe the patient with dry garments.

Curative Treatment.—In all cases, unless the bowels happen to be very loose, small doses of calomel and soda, say one-fourth grain of the former and one grain of the latter, should be taken for every hour for eight or ten doses, and if the bowels do not move freely by the next morning, follow it up with a Seidlitz powder, glass of Hunyadi water or a dose of magnesium sulphate taken one hour before breakfast. A good many drugs have been at various times recommended in the treatment of malarial fevers; of these the derivatives of Cinchona bark hold first place. Quinine or its salts, the sulphate, bi-sulphate, hydrobromide and hydrochloride are probably most frequently used, but quinidine, cinchonine and cinchonidine are also very valuable at times in the treatment. The daily dose of quinine and its salts necessary to break up an attack of intermittent fever in an adult will vary from 20 to 30 grains in twenty-four hours. Twenty grains a day, however, usually suffices. Quinidine, cinchonine and cinchonidine require a larger dose to produce the same effect, about one-third larger than the dose of the quinine salts, and a daily dose of 30 grains.

Administration of Quinine.—(1.) By the mouth. This is the common mode of administering the drug. It may be given in solution, such as, quinine sulphate 1 dram, acid sulphuric dilute 1 dram and syrup of orange q. s. ad 4 ounces; dose, 1 dram in water every two hours. This is, however, a nauseous, disagreeable mixture. Quinine should always be given in capsules where it is possible, and the hydrobromide and hydrochloride being the most soluble and containing the largest percentage of quinine should have the preference. The bi-sulphate is also a valuable salt, although it is more irritating to the gastric mucosa than the above mentioned salts and not containing as much quinine requires that it be given in a larger dose. Quinine and quinine sulphate may also be given suspended in heavy syrups, such as syrups of chocolate, yerba santa or licorice, 2 grains to each dram of the syrup. If pills or tablets of the drug are used they should be freshly prepared, as when old they may be almost insoluble and therefore void of therapeutic action. If the sulphate of quinine is used, a good plan is to have four or five drops of dilute hydrochloric acid in water after each dose, to aid its solution Twenty grains administered during the 24 hours usually suffices to abort an attack. However, some cases require larger doses and in tropical countries a daily dose of from 40 to 60 grains is frequently necessary. It has appeared to me that one-fourth to one-half of a grain of powdered capsicum or one or two grains of powdered ginger added to each dose, has markedly aided its therapeutic action. In the administration of the drug it will be found a good plan to give small doses of two or three grains every two hours after the fever has subsided, but for several hours before the next chill is due to give full doses of the drug, say four grains every hour for three or four doses, so that the system will be thoroughly charged with the drug at least two hours before the chill is due, for the plasmodium is most susceptible to destruction at the time of sporulation.

(2.) By Hypodermic Injection: (a) Intramuscular Injection.—This method affords one of the best means of combating the severe or malignant type of the disease. The injection should be made with a good sized hypodermic syringe holding one or two drams, which should be thoroughly sterilized by boiling before using. The skin over the part to be injected should be carefully cleansed, first with soap and warm water and afterwards with alcohol. The site of injection should preferably be the gluteal region (buttocks) and the injection made deep or intramuscular and not subcutaneous. As a rule injections should not be repeated more often than twice a day; however, in severe and pernicious forms of the disease it may be necessary to give them several times a day. If given in the intermittent form with chills, injections are best given two hours before the chill is due. A new spot should be chosen for each injection and the part gently massaged afterwards to promote the absorption of the drug. The salts of quinine most suitable for hypodermic use are the hydrobromide, hydrochloride, dihydrochloride, bi-muriate with urea and the bi-sulphate, as these are the most soluble. The following formulas are to be recommended for hypodermic use:

R.
Quinine hydrochloride ...... .50 to 1.00 (7½ to 15 grs.)
Alcohol ........................... 1.00 (15 minims)
Glycerine ......................... 1.00 (15 minims)
Aquae (warm sterile)
q. s. ad ........................... 4.00 (1 drachm)
M.
Signa: To be used at one injection.
Or

R.
Quinine hydrochloride .................. 65 (10 grs.)
Alcohol ........................... 1.00 (15 minims)
Glycerine ......................... 1.00 (15 minims)
Aquae (warm sterile)
q. s. ad ........................... 4.00 (1 drachm)
M.
Signa: To be used at one injection.

(In preparing these solutions, dissolve the quinine in 95 per cent. alcohol first, then add the glycerine and water quantity sufficient to make one dram. They should be perfectly clear before using.)—*St. Louis Medical Review.*

---

Nothing In It.—"Now, Tommy," said Mrs. Bull, "I want you to be good while I'm out."

"I'll be good for a nickel," replied Tommy.

"Tommy," she said, "I want you to remember that you can not be a son of mine unless you are good for nothing.—*Louisville Courier-Journal.*

### A STORM CENTER IN SPOKANE.

A tolerably vivid imagination can picture the consternation that would possess the community if the physicians and surgeons were to suddenly retire from business. Go to our great hospitals, where the doctor's visit is regarded by the suffering patients as one of the happiest moments of the day, and think what the feeling would be in such institutions if the footfall of the physician were to be heard no more. Walk into the waiting rooms of the specialists of any large city, and see the dejected looks upon the patients as their eyes would greet a sign—"permanently retired from business." And yet, ministering as they do to the sufferings of humanity, the physicians and surgeons are liable at any time to be reviled by irresponsible reporters on the daily newspapers, who are always looking for something sensational. At Spokane, just now, one can find in the headlines of the sensational newspapers the medical men of that city referred to as "dopers and carvers." This is even more contemptible and unworthy of decent journalism than to call a dentist a "tooth carpenter" although this is bad enough. The Spokane trouble arises over a very proper crusade that is being made against that particular form of quackery called "chiropractic" operators, who are writing letters to the papers, and in other ways expressing their disapprobation of the laws of the state, and of those who are intent upon enforcing them. One of these correspondents says that it would be as reasonable to suppress their particular form of nerve massage as to suppress the shampooer in a Turkish bath, who, for pay, endeavors to benefit his visitor. The sensible part of the public, which, unfortunately, includes but a small proportion of mortals, can see the fallacy of this reasoning. But many of the newspapers are ever ready to give encouragement to this class of fakirs, partly because they contribute their money to the advertising columns. Spokane seems to be an exceptional storm center just at this time for this sort of excitement, and we wish for strength of arm to the law of the State of Washington, and to the enforcers thereof who live at Spokane.—*Medical Sentinel,*

### SYPHILIS IN CHILDREN.

Weiss states that the number of cases of inherited syphilis far outnumbers the cases acquired. He divides the modes of transmission into three classes.

1. Direct infection of the healthy mother by the diseased father having florid secondary symptoms causing in the mother the same kind of lesions. The child so conceived usually dies in utero and is aborted. 2. Direct transmission of syphilitic taint through the infection sperm of the father, the mother being healthy. In such cases, the mother may remain well and even the child may escape. The mother may become immune through absorption of toxins elaborated by the fetus. When the infection is on the part of the mother with a diseased ovule the taint is more disastrous to the child than paternal heredity. This condition is rare. 3. Infection of the fetus when the parents were healthy at the time of impregnation and infection of the mother takes place during pregnancy. By treatment of the parents or by natural attenuation, viable healthy children may be born and these are immune against the acquisition of syphilis.—*N. Y. State Journal of Medicine.*

### THE MANAGEMENT OF THE BOWELS IN TYPHOID FEVER.

The specific inflammation caused by this disease is located in the lower part of the small intestine. The result of inflammation in the intestine is diarrhea, consequently diarrhea is a symptom of this disease and is Nature's method of removing undigested food and the products of inflammation. Hence constipation is a thing not desired, and should be prevented when it occurs in typhoid fever. On the other hand, very frequent, or even a number of movements a day should be prevented; consequently we should aim to have a daily complete evacuation of the bowels by artificial means.

It is always a good plan at the beginning of this fever, as in others. to administer one dose of calomel, as:

| R. | gm. | |
|---|---|---|
| Hydrargyri chloridi mitis .................... | \|30 or | gr.v |
| Sodii bicarbonatis .. .. ...................... | 1\| | gr.xv |

M. et fac chartulam 1.

Sig.: Take at once.

The dose of calomel may be repeated in forty-eight hours, if deemed advisable, but later in the disease calomel is best not given. Castor oil in capsules, or in clear coffee, or with some effervescing solution, as Vichy, is strongly recommended, in small doses, as a laxative, and if the patient's stomach does not repudiate it, is perhaps one of the best. A method found very satisfactory is to give a laxative, and one that does not cause much peristalsis and causes no irritation, on every alternate day. Some simple saline, as Rochelle salt, Carlsbad salts, Hunyadi, or any simple saline aperient, in the dose found sufficient, is very satisfactory. The advantages of the salines are that they clean the stomach, aid the liver by passing along all the old bile, and relieve the congestion of the portal circulation, all of which is more or less needed in this long-continued sickness.

On the alternate day, the day on which the laxative is not administered, the bowels should be moved by a small enema consisting of a tablespoonful of glycerin and two tablespoonfuls of water.

The nurse should be supplied with very soluble tablets each containing 1-10 grain morphin sulphate with instructions to administer one of these tablets after two large movements or three small ones, so that the laxative may not cause a troublesome diarrhea. This amount of morphin is generally sufficient to stop the action of the bowels for a number of hours. If it is not successful, the dose could be repeated.

By this method toxemia is diminished, fermentation and gas distension are also prevented, the tongue remains moist and not heavily coated, and the danger from hemorrhage and perforation, which is so much more likely to occur from distension, is reduced to a minimum. The so-called typhoid symptoms are also less by this management of the disease, i.e., the dilirium, stupor, and symptoms of nervous irritation as subsultus and carphologia are markedly less in evidence. Of course it should be understood that intestinal hemorrhage, symptoms of peritonitis, great circulatory weakness, or very low temperature would preclude the administration of any laxative until such symptoms had abated.

Whether or not so-called bowel antiseptics should be administered must be decided by the belief of each individual physician. There is no question that some drugs will produce a certain amount of antisepsis in the upper part of the bowel. How far such action extends it is impossible to determine. It is absurd to suppose that bowel antiseptics could be carried to the region of inflammation in typhoid fever. It is just as absurd for the laboratory scientist to declare that because fecal matter can never be rendered antiseptic by administration of drugs by the mouth, that bowel antiseptics are of no value. Consequently, it seems best many times to administer several doses of a drug that is known to diminish fermentation and at least to inhibit an excessive growth of germs, and possibly prevent the colon bacillus and typhoid bacillus from migrating to the upper part of the intestine. Various intestinal antiseptics have been suggested. The best of these are probably phenylis salicylas (salol), betanaphthol bismuth, thymol, various salicylic acid combinations, various benzoates, various phenol combinations, and various proxid of hydrogen salts. It is not necessary to discuss the varying values or powers of these drugs. It is certainly inexcusable to use any drug in this disease that would interfere with the function of any organ or that would deteriorate the blood.

Provided that the kidneys show no congestion and the urine contains no albumin, salol in small doses is one of the most efficient drugs to administer for this purpose. It does not disturb the stomach and it is broken up in the upper part of the intestine into salicylic acid and phenol. If this drug is selected, it should be administered as follows:

R. gm.
Phenylis salicylatis .... ........ ........ .... ....4 or 3i
Fac capsulas 20.
Sig.:One capsule every six hours.

If at any time the urine becomes very concentrated or albumin is found, or the urine assumes a dark color, the salol should be stopped.

It has been claimed that dilute hydrochloric acid administered several times a day, given very well diluted, not only is an aid to stomach digestion, but has a specific good action in typhoid fever. Certain it is that dilute hydrochloric acid is a stimulant to the intestinal secretions.

R. gm.
Acidi hydrochlorici diluti .... .... .... .... ....25 or 3i
Sig.: Ten drops in half a glass of water every six hours.—*Jour. A. M. A.. Therapeutics,* Oct 12th, 1907.

